# How Does the Burden of Respiratory Syncytial Virus Compare to Influenza in Spanish Adults?

**DOI:** 10.1111/irv.13341

**Published:** 2024-06-24

**Authors:** Federico Martinón‐Torres, Clara Gutierrez, Ana Cáceres, Karin Weber, Antoni Torres

**Affiliations:** ^1^ Translational Pediatrics and Infectious Diseases Hospital Clínico Universitario de Santiago de Compostela Santiago de Compostela Spain; ^2^ Genetics, Vaccines and Pediatric Infectious Diseases Research Group, Instituto de Investigación Sanitaria de Santiago Universidad de Santiago Santiago de Compostela Spain; ^3^ Consorcio Centro de Investigación Biomédica en Red de Enfermedades Respiratorias (CIBERES) Instituto de Salud Carlos III Madrid Spain; ^4^ Infectious Diseases and Vaccines Janssen‐Cilag Madrid Spain; ^5^ Global Medical Affairs IDV Janssen‐Cilag Vienna Austria; ^6^ Department of Pneumonology Hospital Clinic of Barcelona Barcelona Spain; ^7^ Pulmonology Department August Pi i Sunyer Biomedical Research Institute (IDIBAPS) Barcelona Spain; ^8^ ICREA Academia, Life and Medical Sciences Universitat de Barcelona Barcelona Spain

**Keywords:** burden of disease, epidemiology, hospitalization, human, influenza, respiratory syncytial virus

## Abstract

**Background:**

Respiratory syncytial virus (RSV) and influenza infections cause significant annual morbidity and mortality worldwide in at‐risk populations. This study is aimed at assessing hospital burden and healthcare resource utilization (HRU) of RSV and influenza in adults in Spain.

**Methods:**

Data were obtained from the Projected Hospitalisation Database of inpatient episodes (ages: younger adults 18–50 and 51–64 years; older adults 65–74, 75–84, and ≥ 85 years) during 2015, 2017, and 2018 in Spanish public hospitals. Incidence, mean hospitalization, and HRU assessments, including length of stay (LOS), intensive care unit (ICU) usage, and age‐standardized mortality rates, were collected and stratified by age group, with analyses focusing on the adult population (≥ 18 years old).

**Results:**

Mean hospitalization rate in the population across all years was lower in individuals with RSV versus influenza (7.2/100,000 vs. 49.7/100,000 individuals). ICU admissions and median LOS were similar by age group for both viruses. Age‐standardized mortality was 6.3/100,000 individuals and 6.1/100,000 individuals in patients with RSV and influenza, respectively, and mortality rates were similar in older adults (≥ 65 years) for both viruses.

**Conclusions:**

RSV and influenza infection were associated with considerable HRU. There is a substantial disease burden for RSV infection in older adults ≥ 65 years. While RSV hospitalization rates in adults reported here appeared lower than influenza, RSV is still underdiagnosed in the hospital setting and its incidence might be similar to, or higher than, influenza.

## 1 Introduction

Viral respiratory tract infections (RTIs) pose a significant public health problem due to their frequency and ease of transmission [1, 2]. Influenza and respiratory syncytial virus (RSV) are among the leading causes of acute lower RTI [3]; epidemics frequently overlap [4] and cause substantial global morbidity and mortality [3, 5, 6, 7]. During winter, incidence and severity of RTIs peak and the burden of seasonal influenza and RSV contributes to substantial healthcare resource utilization (HRU) globally; however, estimating the true burden is challenging [5, 7, 8]. There are wide variations in excess mortality estimates between countries, seasons, and years for influenza, influenced by a mismatch of circulating viral and seasonal vaccine strains, environmental temperatures, vaccination coverage, and population demographics [6, 9]. The Commission of European Communities in 2009 estimated the direct and indirect costs of an influenza epidemic in industrialized countries to be €56.7 million/1,000,000 people, while the annual cost of hospital admissions estimate was €11.5 billion [10, 11]. Global pandemics are unpredictable and further amplify HRU; for example, hospitalization costs due to the 2009 influenza pandemic in Spain were estimated at €35.4 million [12].

Although not as well recognized as influenza, RSV is among the most common pathogens in acute lower RTI, which remains a leading cause of infant and child hospitalization globally [5, 13, 14]. In adults, its impact has only recently gained appreciation with a disease burden possibly similar to that of influenza [15, 16]. Similar observation was made in Spain, especially in elderly and other high‐risk (e.g., immunocompromised) patients [17]. However, this burden may still be under‐recognized for a lack of disease awareness among clinicians and limited testing [18, 19, 20].

Although nearly all adults will have been infected with RSV during their lifetime [21], severe lower RTI can occur, particularly among adults ≥ 65 years and/or those with comorbidities, resulting in an increased risk of complications, respiratory failure, prolonged hospitalizations, and mortality [7, 9, 22, 23]. Unlike influenza, there is no licensed direct‐acting RSV treatment available to reduce the burden on HRU, and the management of RSV is currently centered around disease prevention and primary supportive care [24]. Initiatives have only recently been taken to enhance RSV awareness and improve diagnostic practices within the Spanish health system [25]. Additionally, RSV vaccines for use during pregnancy and for older adults have been recently approved and are effective at preventing severe lower RTI [26, 27, 28], which may reduce RSV‐associated HRU.

To support future developments of prevention programs and optimize resource allocation for upcoming influenza and RSV seasons, the aim of this study was to further understand the epidemiology of these pathogens, focusing on disease burden and associated HRU in the adult population in Spanish inpatient settings.

## 2 Methods

A cross‐sectional study was conducted using data from the Projected Hospitalisation Database (PHDB), arising from extrapolation of the Minimum Basic Data Sets (MBDSs) from 189 Spanish public hospitals, to obtain results throughout the public hospital system.

The MBDS collects anonymous inpatient episode information, which includes demographic, administrative, and clinical data, and records diagnosis and procedure codes. Collected data were sent to IASIST–IQVIA, a healthcare information company, to independently build a benchmarking database and conduct statistical studies. IASIST–IQVIA used a statistical projection method to assess the potential of hospitals to be excluded from this database (Figure S1). Each hospital was classified using stratification variables, such as geographical area or services provided. Of all the possible variables, the group of hospitals was represented by a subset of them, defined as the Minimum Stratification Unit (MSU). The average values per MSU were calculated and used to estimate the values for each hospital across the country. To measure reliability, the correlation between the estimated and actual values was calculated for each indicator. By using this projection method and determining the MSU, it was established that considering ~55% of total hospital admissions in Spain was representative of the whole patient population.

While the database includes all patients discharged with RSV or influenza A between 1 January and 31 December of 2015, 2017, and 2018, the reference population was the entire Spanish population for each year, obtained from the National Institute of Statistics (INE). As the population data were defined either at the beginning or the middle of each year (1 January in 2015 and 2017; 31 July in 2018), incidence was calculated per 100,000 individuals. Data from 2016 were excluded due to the transition from ICD‐9‐CM to ICD‐10‐ES, making data unreliable for analysis.

Coding for RSV and influenza hospitalizations is presented in Tables S1 and S2. Patient characteristics and HRU (Table S3) were calculated for the entire period. Data were stratified by age (younger adults: 18–50 and 51–64 years; older adults: 65–74, 75–84, and ≥ 85 years).

Data were analyzed according to the presence/absence of comorbidities (any predefined condition; Table S4). Readmissions were defined as respiratory‐related admissions within 30 days after discharge for an RSV or influenza event, using ICD‐9‐CM and ICD‐10‐ES codes (Table S5). Complications were identified using ICD‐9‐CM and ICD‐10‐ES codes (Table S6).

### 2.1 Statistical Analysis

Mean hospitalization rate was defined as the average number of discharged hospitalization cases during the period divided by the average Spanish population at the beginning or midpoint of the study period. Length of stay (LOS) was the number of days between admission and discharge. All‐cause in‐hospital mortality was the number of patients deceased at discharge divided by the number of inpatient episodes. Complications (occurring after index hospitalization), intensive care unit (ICU) admissions, respiratory readmissions, and mechanical ventilation (MV) use were defined as the number of episodes with the event divided by the number of hospitalizations. LOS and mortality were also calculated in patients who required ICU care during hospitalization. Age‐standardized mortality rates were calculated using Spanish population figures from the INE with the European Standard Population 2013 as the reference. Rates were expressed per 100,000 individuals. For each categorical variable, frequency and percentage were calculated. All analyses were performed using IBM SPSS Statistics 21.0. Dispersion measures were estimated using interquartile ranges (IQRs). Statistical significance was not reported, as no *P* values were calculated.

This study was approved by the ethics committee of the “Hospital Clínic de Barcelona” (registration number: HCB/2020/0738). Patient information was available for scientific purposes while guaranteeing anonymity and preserving personal identification data according to current data protection laws.

## 3 Results

### 3.1 Incidence of Hospitalizations

Across 2015, 2017, and 2018, a mean annual 2750 RSV and 19,013 influenza infections in adults aged ≥ 18 years resulting in hospitalization were reported. The overall mean incidence rate of infection in the population was 7.2/100,000 individuals for RSV and 49.7/100,000 individuals for influenza (Table 1).

**TABLE 1:** Mean incidence rate of hospitalizations per 100,000 individuals for respiratory syncytial virus and influenza in Spain (2015, 2017, and 2018).

	RSV		Influenza	
	Mean N	Incidence rate	Mean N	Incidence rate
Younger adults	665	2.3	4759	16.2
18–50 years	227	1.1	1907	9.2
51–64 years	438	5.1	2852	33.2
Older adults	2085	23.7	14,254	161.8
65–74 years	480	10.8	3484	78.3
75–84 years	796	26.7	5596	187.9
≥ 85 years	809	55.9	5174	363.5
Total	2750	7.2	19,013	49.7

Abbreviations: Mean N, mean number of cases for the study period; RSV, respiratory syncytial virus.

Age was positively associated with the incidence of hospitalization in the population for RSV and influenza (Table 1), with the highest rates observed in adults ≥ 85 years (RSV: 55.9/100,000 individuals; influenza: 363.5/100,000 individuals). When stratified by age, hospitalization for influenza was significantly higher than for RSV in adults ≥ 65 years (*p* < 0.05; Table 1).

### 3.2 LOS and Comorbidities

Median LOS was consistently longer across all age groups for RSV versus influenza (Table 2) and peaked in adults 51–64 years (8.5 vs. 6.3 days, respectively).

**TABLE 2:** Length of stay in patients infected with respiratory syncytial virus or influenza stratified by age group and presence of comorbidities in Spain (2015, 2017, and 2018).

		Overall		With comorbidities		Without comorbidities	
	Comorbidity incidence (%)	N	LOS, days (IQR)	n (%)	LOS, days (IQR)	n (%)	LOS, days (IQR)
RSV							
Younger adults		665	8.0	480	8.9	185	7.1
18–50 years	62.1	227	8.0 (3–12)	137 (60.4)	9.3 (3–13)	90 (39.6)	6.3 (3–9)
51–64 years	80.4	438	8.5 (4–14)	343 (78.3)	8.8 (4–13)	95 (21.7)	7.8 (3–9)
Older adults		2085	7.6	1139	11.6	137	10.5
65–74 years	84.0	480	7.7 (4–11)	403 (84.0)	7.7 (4–11)	77 (16.0)	7.7 (3–9)
75–84 years	92.4	796	7.3 (4–11)	736 (92.5)	7.3 (4–12)	60 (7.5)	6.7 (5–15)
≥ 85 years	92.0	809	6.7 (4–9)	754 (93.2)	6.3 (4–9)	55 (6.8)	8.0 (4–12)
Influenza							
Younger adults		4759	5.6	2847	6.3	1912	4.6
18–50 years	44.7	1907	4.7 (3–9)	846 (44.4)	5.5 (3–10)	1061 (55.6)	4.3 (3–8)
51–64 years	70.7	2852	6.3 (4–11)	2001 (70.2)	6.7 (4–12)	851 (29.8)	5.0 (3–10)
Older adults		14,254	6.3	12,540	6.5	1715	5.6
65–74 years	83.8	3484	6.3 (4–10)	2887 (82.9)	6.3 (4–11)	597 (17.1)	5.3 (3–9)
75–84 years	89.4	5596	6.3 (4–11)	4964 (88.7)	6.7 (4–11)	633 (11.3)	5.3 (3–10)
≥ 85 years	91.2	5174	6.3 (4–10)	4689 (90.6)	6.3 (4–10)	485 (9.4)	6.3 (3–10)

*Note:* Comorbidities were considered as the presence of any predefined conditions (Table S4).

Abbreviations: IQR, interquartile range; LOS, length of stay; RSV, respiratory syncytial virus.

The proportion of patients with comorbidities generally increased with age in both groups. Among adults 18–64 years, hospitalizations of patients with comorbidities were more frequent in the RSV than the influenza group; while among adults ≥ 65 years, the frequency was slightly higher in the RSV than the influenza group (Table 2). The age group with the highest proportion of patients with comorbidities was 75–84 years in the RSV (92.5%) and ≥ 85 years in the influenza group (90.6%; Table 2).

Median LOS by age group was generally longer in patients with comorbidities than in those without (Table 2) and was consistently longer in the RSV than the influenza group when stratifying patients by those with or without comorbidities, across all age categories, except those ≥ 85 years (Table 2).

### 3.3 Complications, ICU, and MV Requirements

There was a trend of complications arising from hospitalization increasing substantially with age (Table 3). The proportion of patients with complications was similar in both groups and across all age categories, although acute kidney disease was more frequent in the RSV than the influenza group (Table S7). The most commonly reported complications in the RSV and influenza groups (respectively) were respiratory failure (39%; 40%), exacerbation of pulmonary disease (16.9%; 18.7%), and exacerbation of chronic kidney disease (16.8%; influenza; Table S7).

**TABLE 3:** Mortality, complications, related readmissions, and invasive mechanical ventilation in patients infected with respiratory syncytial virus or influenza by age group in Spain (2015, 2016, and 2018).

	N	Mortality (%)	Complications (%)	Readmissions (%)	Invasive MV (%)
RSV					
Younger adults	665	4.6	60.2	7.9	6.9
18–50 years	227	4.1	47.0	7.9	5.6
51–64 years	438	4.8	67.1	7.9	7.5
Older adults	2085	7.2	79.2	11.2	2.9
65–74 years	480	5.0	74.6	8.3	5.1
75–84 years	796	7.5	81.4	10.2	3.7
≥ 85 years	809	8.2	79.7	13.8	0.8
Influenza					
Younger adults	4759	3.5	57.8	4.8	6.0
18–50 years	1907	2.2	46.5	3.7	5.6
51–64 years	2852	4.4	65.3	5.6	6.3
Older adults	14,254	7.3	76.5	8.3	2.6
65–74 years	3484	4.8	73.7	6.8	5.1
75–84 years	5597	6.3	77.1	8.7	3.0
≥ 85 years	5174	10.2	77.8	9.0	0.4

*Note:* Proportions were calculated based on the average number of cases over the study period (2015, 2017, 2018) for each category.

Abbreviations: MV, mechanical ventilation; RSV, respiratory syncytial virus.

Rates of ICU admission were slightly higher in the RSV versus the influenza group (Table 4), with the highest rates observed in individuals aged 51–64 years (RSV: 15.1%; influenza: 12.7%). For both virus groups, time spent in the ICU comprised a considerable portion of overall median LOS across age groups (Table 4).

**TABLE 4:** Length of stay and mortality of patients infected with respiratory syncytial virus or influenza requiring intensive care during their hospital stay in Spain (2015, 2017, and 2018).

	N	ICU (%)	Median LOS in hospital of patients requiring ICU, days (IQR)	Median LOS in ICU only, days (IQR)	Mortality (%)	Mortality in ICU (%)
RSV						
Younger adults	83	14.5	18 (12–42)	7.2 (3–26)	21.0	17.8
18–50 years	30	13.4	16 (13–40)	3.7 (2–4)	16.9	15.1
51–64 years	53	15.1	19 (15–37)	7.5 (4–26)	23.3	19.4
Older adults	111	10.4	16 (12–21)	5 (3–13)	38.0	23.0
65–74 years	49	11.5	17 (12–22)	5.5 (3–13)	19.5	14.7
75–84 years	52	11.1	22 (15–21)	5 (4–9)	54.2	27.9
≥ 85 years	10	1.3	14 (12–18)	6 (2–41)	40.5	38.1
Influenza						
Younger adults	585	12.7	17 (12–19)	8 (4–19)	18.8	16.5
18–50 years	232	12.6	16 (13–19)	9 (3–19)	14.4	11.8
51–64 years	353	12.7	17 (12–19)	8 (4–19)	21.7	19.6
Older adults	744	7.9	16 (13–19)	6 (6–14)	31.4	23.0
65–74 years	344	10.4	18 (14–23)	7 (3–15)	28.4	21.8
75–84 years	338	6.6	16 (13–18)	6 (3–13)	32.6	22.5
≥ 85 years	62	1.4	12 (9–15)	5 (2–13)	41.8	32.6

*Note: N* represents the average number of individuals requiring ICU care over the study period. ICU % represents the total number of individuals using ICU over the total number of cases for the age group considered. Mortality represents deaths occurring among individuals requiring ICU care. Mortality in the ICU refers to deaths occurring within the ICU.

Abbreviations: ICU, intensive care unit; LOS, length of stay; RSV, respiratory syncytial virus.

Rates of invasive MV usage by age group were generally higher in the RSV than the influenza group (Table 3). These rates were highest in the 51–64 age group for both RSV (7.5%) and influenza (6.3%), after which the rates of invasive MV decreased (most noticeably in patients ≥ 85 years).

### 3.4 Readmissions and Mortality

Related readmissions after initial hospitalization generally increased with age and were highest in older adults (≥ 65 years) for both virus groups, peaking at 13.8% in the RSV and 9% in the influenza group in adults ≥ 85 years. Readmissions were consistently higher for RSV than for influenza across all age categories (Table 3).

The trend for overall mortality among patients increased with age in both groups, peaking in patients aged ≥ 85 years (RSV: 8.2%; influenza: 10.2%; Table 3). Mortality rates in the RSV group aged 18–50 years were nearly double that found in the influenza group (4.1% vs. 2.2%; Table 3).

Mortality rate among patients who required intensive care was similar in both virus groups, except for that of patients aged 75–84 years, which was considerably higher in the RSV (54.2%) than the influenza group (32.6%). Mortality rates among patients admitted to the ICU were consistently higher than the overall mortality rate (Table 4). At a whole population level, age‐standardized mortality rates for in‐hospital mortality were similar between groups (RSV: 6.3/100,000 individuals; influenza: 6.1/100,000 individuals). In older adults (≥ 65 years), the age‐standardized mortality rates were 7.0/100,000 individuals and 7.1/100,000 individuals in the RSV and influenza groups, respectively.

## 4 Discussion

This cohort study of adults hospitalized with RSV or influenza in Spain in 2015, 2017, and 2018 demonstrated substantial HRU associated with both viruses that increases with age, especially in those aged ≥ 65 years. Similar trends between RSV and influenza were seen for comorbidities, complications, intensive care use, and mortality. However, invasive MV usage and readmission rates were consistently higher, and median LOS was consistently longer for patients with RSV versus influenza.

Hospitalizations due to RSV or influenza were reported in similar studies with varying incidence rates [5, 29, 30, 31]. Wide‐ranging differences between countries, seasons, and years can affect the incidence of influenza, attributed to climatic and environmental factors, seasonal viral strains, vaccination coverage, and population demographics [6, 9]. Variations in the RSV season have also been described [32, 33], with the Spanish season differing to the typical biannual rhythm found in many other European countries, characterized by a stable epidemic peaking between Weeks 52 and 1, and circulating 2–8 weeks earlier than influenza [34].

Despite variations in the hospitalization incidence and lower disease incidence for RSV versus influenza, this study demonstrates the increasingly recognized RSV disease burden in older adults [29, 35]. The lower estimates of RSV disease burden versus influenza may be due to a lower index of clinical suspicion and fewer diagnostic tests applied. Increased use of tests such as polymerase chain reaction panels, which can accurately distinguish between many pathogens with similar symptoms, could lead to further detection and more precise estimates of the true RSV incidence.

Importantly, we also report an underappreciated RSV disease burden in younger adults. The highest rates of ICU admission, invasive MV usage, and longest median LOS were observed in patients aged 51–64 years, with higher rates observed for patients with RSV. In this age demographic, the frequency of hospitalizations of patients with comorbidities was also higher in the RSV than the influenza group. Additionally, overall mortality rates in the RSV group among adults aged 18–50 years were nearly double that found in the influenza group. While in both groups, the rates of invasive MV were lower in adults aged ≥ 65 years versus younger adults; this is likely due to a conservative approach owing to the higher risk of invasive procedures in older adults. This study demonstrated an association with the presence of comorbidities in adults hospitalized with RSV and poor outcomes, such as complications and mortality, regardless of age. These data add to growing body of evidence of the burden of RSV in high‐risk adults [7, 8, 36] and highlight the importance of effective vaccines in this population.

Barriers remain to reducing RSV disease burden and associated HRU, such as a lack of disease awareness and effective treatments, particularly in adult populations [18]. Furthermore, testing for RSV is not widely used in adults. When limited testing capability is combined with the similarity of symptoms between RSV and other respiratory pathogens, clinicians may not suspect RSV as the primary cause, thus minimizing its true impact [19, 20]. Diagnostic challenges are currently compounded by licensed treatments (ribavirin, palivizumab, and nirsevimab) only being offered to children under limited circumstances [37, 38]. The availability of newly approved vaccines against RSV for older adults potentially represents a significant paradigm shift in adult RSV. In contrast, there is a range of licensed vaccines for influenza, as well as antivirals, including oseltamivir and baloxavir marboxil, which were the main licensed drugs for both treatment and postexposure prophylaxis during this study.

To our knowledge, this is one of the first reports of Spanish national estimates of in‐hospital mortality for RSV, ranging between 4.1% and 4.8% for adults aged 18–50 and 51–64 years, respectively, and between 5% and 8.2% for older adults aged ≥ 65 years; this was similar to influenza for the same age groups and was markedly increased in those requiring ICU admission. This is consistent with previously reported national data where the mortality rate of RSV was similar to that of influenza, particularly in adults aged ≥ 65 years [39, 40, 41]. Our results are also consistent with a report from Spain during 2016–2019 based on data from the Spanish Ministry of Health and INE, where an age‐dependent increase in the mortality rates in adults was observed, although to a lower extent than in our study [42].

The COVID‐19 pandemic has raised awareness of the major risks of respiratory viruses to public health and potential burden on healthcare systems. There is an opportunity to address RSV‐associated disparities in older adults, through the implementation of vaccination programs, alongside development of antiviral treatments. For example, in a study of influenza‐related hospitalization costs in adults aged ≥ 65 years in Spain, vaccination reportedly reduced ICU admissions, rates of MV, and the degree of dependence on hospital services [43]. This is despite the influenza vaccination rate of 54.3% in this age group being short of the 75% goal recommended by the World Health Assembly [44, 45]. Therefore, it is reasonable to suggest that newly available RSV vaccines could offer protection against the severity of RSV‐associated disease, thus reducing the burden on HRU.

### 4.1 Strengths and Limitations

This study used data from 189 Spanish public hospitals, representing ~55% of all Spanish national healthcare system episodes annually. Since episodes were linked directly to the intensive care database, ICU usage could be accurately described. Furthermore, because this database is a sample from the Spanish national healthcare system, results can be generalized to the entire population. A further strength of this study is the wide age range of individuals included in the study population. These data may guide policy‐makers to target specific groups for potential future intervention programs, such as younger adults with comorbidities.

Regarding limitations, infections were identified based on ICD coding, which are subject to misclassification bias, potential coding errors, and missing information. Identifying cases through ICD coding without diagnostic testing may underestimate true disease burden, especially for RSV as testing is not routine. Data derived from medical records may be subject to human error or differences in recording practices. Additionally, the overall number of tests performed in this study is unknown, making it difficult to interpret the true incidence of confirmed RSV cases. This study is also only representative of the hospital setting and may underestimate the true mortality impact from both infections, which may be best evaluated 30‐day postdischarge.

RSV and influenza infections are rarely confirmed by laboratory diagnosis, and episodes or deaths might be attributed to comorbid conditions or secondary infections. Some age groups spanned larger ranges than others (particularly the 18–50 age group), which may have skewed the results for certain attributes in these groups. No adjustments were made for seasonality, vaccine status, or other variables known to impact hospitalization for both groups.

## 5 Conclusion

These data highlight the substantial disease burden of RSV and influenza in older adults (≥ 65 years). While RSV hospitalization rates appeared lower than influenza, RSV remains underdiagnosed in the hospital setting and its incidence might be similar to that of influenza. This study also demonstrated an underappreciated disease burden for RSV in younger adults, and these data may inform policy‐makers in the development of potential future intervention programs to target specific at‐risk groups. Overall, there is an urgent need for widespread RSV vaccination programs for older adults, effective vaccines for other high‐risk adults, and effective therapeutic interventions for RSV, as well as increased awareness from healthcare practitioners regarding RSV burden in the adult population.

## Author Contributions

**Federico Martinón‐Torres:** conceptualization, methodology, writing—review and editing. **Clara Gutierrez:** project administration, supervision, writing—review and editing. **Ana Cáceres:** project administration, supervision, writing—review and editing. **Karin Weber:** conceptualization, methodology, supervision, writing—original draft, writing—review and editing. **Antoni Torres:** conceptualization, methodology, writing—review and editing.

## Ethics Statement

This study was approved by the ethics committee of the “Hospital Clínic de Barcelona” (registration number: HCB/2020/0738). Patient information was available for scientific purposes while guaranteeing anonymity and preserving personal identification data according to current data protection laws.

## Conflicts of Interest

F. Martinón‐Torres received honoraria from GSK group of companies, Biofabri, Pfizer Inc., Sanofi Pasteur, MSD, Seqirus, and Janssen for taking part in advisory boards and expert meetings and for acting as a speaker in congresses outside the scope of the submitted work. F. Martinón‐Torres has also acted as principal investigator in randomized controlled trials of the above‐mentioned companies as well as Ablynx, Gilead, Regeneron, Roche, Abbott, Novavax, and MedImmune, with honoraria paid to his institution. C. Gutierrez and A. Cáceres are employees of Janssen Pharmaceuticals, while K. Weber previously worked for this company, and may be Johnson & Johnson stockholders. A. Torres has received honoraria from lectures or advisory boards from Pfizer, MSD, Janssen, and bioMérieux.

### Peer Review

The peer review history for this article is available at https://www.webofscience.com/api/gateway/wos/peer‐review/10.1111/irv.13341.

## Supporting information


**FIGURE S1** Statistical projection method to assess the potential of hospitals to be excluded from this study.
**TABLE S1:** Diagnostic codes used to identify RSV patients.
**TABLE S2:** Diagnostic codes used to identify influenza patients.
**TABLE S3:** Other procedure codes used to identify cases of interest.
**TABLE S4:** Diagnostic codes used to identify predefined comorbidities.
**TABLE S5:** Respiratory diagnoses (for analyses of *respiratory* readmission < 30 days after RSV/influenza A admission discharge).
**TABLE S6:** Diagnostic codes used to identify complications.
**TABLE S7:** Complications in patients infected with respiratory syncytial virus or influenza stratified by age group in Spain (2015, 2017, and 2018).

## Data Availability

The data analyzed in this study are not publicly available due to licensing agreement with IQVIA.

## References

[1] P. K. Pattemore and L. C. Jennings , “Epidemiology of Respiratory Infections,” Pediatric Respiratory Medicine (2008): 435–452.

[2] N. H. L. Leung , “Transmissibility and Transmission of Respiratory Viruses,” Nature Reviews. Microbiology 19 (2021): 528–545. 33753932 10.1038/s41579-021-00535-6 PMC7982882

[3] S. Safiri , A. Mahmoodpoor , A. A. Kolahi , et al., “Global Burden of Lower Respiratory Infections During the Last Three Decades,” Frontiers in Public Health 10 (2023): 1028525. 36699876 10.3389/fpubh.2022.1028525 PMC9869262

[4] M. Chadha , S. Hirve , C. Bancej , et al., “Human Respiratory Syncytial Virus and Influenza Seasonality Patterns‐Early Findings From the WHO Global Respiratory Syncytial Virus Surveillance,” Influenza and Other Respiratory Viruses 14 (2020): 638–646. 32163226 10.1111/irv.12726 PMC7578323

[5] R. T. Stein , L. J. Bont , H. Zar , et al., “Respiratory Syncytial Virus Hospitalization and Mortality: Systematic Review and Meta‐analysis,” Pediatric Pulmonology 52 (2017): 556–569. 27740723 10.1002/ppul.23570 PMC5396299

[6] A. D. Iuliano , K. M. Roguski , H. H. Chang , et al., “Estimates of Global Seasonal Influenza‐Associated Respiratory Mortality: A Modelling Study,” Lancet 391 (2018): 1285–1300. 29248255 10.1016/S0140-6736(17)33293-2 PMC5935243

[7] J. Hartnett , P. Donga , G. Ispas , et al., “Risk Factors and Medical Resource Utilization in US Adults Hospitalized With Influenza or Respiratory Syncytial Virus in the Hospitalized Acute Respiratory Tract Infection Study,” Influenza and Other Respiratory Viruses 16 (2022): 906–915. 35474419 10.1111/irv.12994 PMC9343339

[8] A. R. Falsey , E. E. Walsh , S. House , et al., “Risk Factors and Medical Resource Utilization of Respiratory Syncytial Virus, Human Metapneumovirus and Influenza Related Hospitalizations in Adults – A Global Study During the 2017–2019 Epidemic Seasons (Hospitalized Acute Respiratory Tract Infection [HARTI] Study),” Open Forum Infectious Diseases 8 (2021): ofab491. 35559130 10.1093/ofid/ofab491 PMC9088513

[9] L. S. Vestergaard , J. Nielsen , T. G. Krause , et al., “Excess all‐Cause and Influenza‐Attributable Mortality in Europe, December 2016 to February 2017,” Euro Surveillance 22 (2017): 30506. 28424146 10.2807/1560-7917.ES.2017.22.14.30506 PMC5388126

[10] Commission of the European Communites , “Proposal for a Council Recommendation on Seasonal Influenza Vaccination 2009,” accessed January 19, 2023, https://ec.europa.eu/health/ph_threats/com/Influenza/docs/seasonflu_rec2009_en.pdf.

[11] J. Ryan , Y. Zoellner , B. Gradl , B. Palache , and J. Medema , “Establishing the Health and Economic Impact of Influenza Vaccination Within the European Union 25 Countries,” Vaccine 24 (2006): 6812–6822. 17034909 10.1016/j.vaccine.2006.07.042

[12] C. Rodríguez‐Rieiro , P. Carrasco‐Garrido , V. Hernández‐Barrera , et al., “Pandemic Influenza Hospitalization in Spain (2009): Incidence, In‐Hospital Mortality, Comorbidities and Costs,” Human Vaccines & Immunotherapeutics 8 (2012): 443–447. 22370516 10.4161/hv.18911

[13] F. Martinón‐Torres , M. Carmo , L. Platero , et al., “Clinical and Economic Burden of Respiratory Syncytial Virus in Spanish Children: The BARI Study,” BMC Infectious Diseases 22 (2022): 759. 36175846 10.1186/s12879-022-07745-0 PMC9520861

[14] J. G. Wildenbeest , M. N. Billard , R. P. Zuurbier , et al., “The Burden of Respiratory Syncytial Virus in Healthy Term‐Born Infants in Europe: A Prospective Birth Cohort Study,” The Lancet Respiratory Medicine 11, no. 22 (2022): 414–413. 10.1016/S2213-2600(22)00414-3 PMC9764871 36372082

[15] A. Sharp , M. Minaji , N. Panagiotopoulos , R. Reeves , A. Charlett , and R. Pebody , “Estimating the Burden of Adult Hospital Admissions Due to RSV and Other Respiratory Pathogens in England,” Influenza and Other Respiratory Viruses 16 (2022): 125–131. 34658161 10.1111/irv.12910 PMC8692807

[16] D. M. Fleming , R. J. Taylor , R. L. Lustig , et al., “Modelling Estimates of the Burden of Respiratory Syncytial Virus Infection in Adults and the Elderly in the United Kingdom,” BMC Infectious Diseases 15 (2015): 443. 26497750 10.1186/s12879-015-1218-z PMC4618996

[17] M. Heppe‐Montero , R. Gil‐Prieto , J. Del Diego Salas , et al., “Impact of Respiratory Syncytial Virus and Influenza Virus Infection in the Adult Population in Spain Between 2012 and 2020,” International Journal of Environmental Research and Public Health 19 (2022): 14680. 36429399 10.3390/ijerph192214680 PMC9690810

[18] NFID. National Foundation for Infectious Diseases , “Call to Action: Reducing the Burden of RSV Across the Lifespan 2022,” accessed January 19, 2023, https://www.nfid.org/wp‐content/uploads/2022/04/NFID‐RSV‐Call‐to‐Action.pdf.

[19] N. Lee , E. E. Walsh , I. Sander , et al., “Delayed Diagnosis of Respiratory Syncytial Virus Infections in Hospitalized Adults: Individual Patient Data, Record Review Analysis and Physician Survey in the United States,” The Journal of Infectious Diseases 220 (2019): 969–979. 31070757 10.1093/infdis/jiz236 PMC6688061

[20] E. E. Walsh , D. R. Peterson , and A. R. Falsey , “Is Clinical Recognition of Respiratory Syncytial Virus Infection in Hospitalized Elderly and High‐Risk Adults Possible?” The Journal of Infectious Diseases 195 (2007): 1046–1051. 17330796 10.1086/511986

[21] G. Berbers , L. Mollema , F. van der Klis , G. den Hartog , and R. Schepp , “Antibody Responses to Respiratory Syncytial Virus: A Cross‐Sectional Serosurveillance Study in the Dutch Population Focusing on Infants Younger Than 2 Years,” The Journal of Infectious Diseases 224 (2021): 269–278. 32964923 10.1093/infdis/jiaa483 PMC8280491

[22] L. Bont , P. A. Checchia , B. Fauroux , et al., “Defining the Epidemiology and Burden of Severe Respiratory Syncytial Virus Infection Among Infants and Children in Western Countries,” Infectious Disease and Therapy 5 (2016): 271–298. 10.1007/s40121-016-0123-0 PMC5019979 27480325

[23] N. Lee , G. C. Lui , K. T. Wong , et al., “High Morbidity and Mortality in Adults Hospitalized for Respiratory Syncytial Virus Infections,” Clinical Infectious Diseases 57 (2013): 1069–1077. 23876395 10.1093/cid/cit471

[24] L. S. Eiland , “Respiratory Syncytial Virus: Diagnosis, Treatment and Prevention,” Journal of Pediatric Pharmacology and Therapeutics 14 (2009): 75–85. 10.5863/1551-6776-14.2.75 PMC3461981 23055894

[25] F. Martinón‐Torres , J. A. Navarro‐Alonso , M. Garcés‐Sánchez , and A. Soriano‐Arandes , “The Path Towards Effective Respiratory Syncytial Virus Immunization Policies: Recommended Actions,” Archivos de Bronconeumología 59 (2023): 581–588. 37414639 10.1016/j.arbres.2023.06.006

[26] B. Kampmann , S. A. Madhi , I. Munjal , et al., “Bivalent Prefusion F Vaccine in Pregnancy to Prevent RSV Illness in Infants,” The New England Journal of Medicine 388 (2023): 1451–1464. 37018474 10.1056/NEJMoa2216480

[27] E. E. Walsh , G. Pérez Marc , A. M. Zareba , et al., “Efficacy and Safety of a Bivalent RSV Prefusion F Vaccine in Older Adults,” The New England Journal of Medicine 388 (2023): 1465–1477. 37018468 10.1056/NEJMoa2213836

[28] A. Papi , M. G. Ison , J. M. Langley , et al., “Respiratory Syncytial Virus Prefusion F Protein Vaccine in Older Adults,” The New England Journal of Medicine 16, no. 388 (2023): 595–608. 10.1056/NEJMoa2209604 36791160

[29] S. Tong , C. Amand , A. Kieffer , and M. H. Kyaw , “Incidence of Respiratory Syncytial Virus Related Health Care Utilization in the United States,” Journal of Global Health 10 (2020): 020422. 33110581 10.7189/jogh.10.020422 PMC7568930

[30] J. Oliva , C. Delgado‐Sanz , A. Larrauri , and Spanish Influenza Surveillance S , “Estimating the Burden of Seasonal Influenza in Spain From Surveillance of Mild and Severe Influenza Disease, 2010–2016,” Influenza and Other Respiratory Viruses 12 (2018): 161–170. 28960828 10.1111/irv.12499 PMC5818358

[31] J. Van Summeren , A. Meijer , G. Aspelund , et al., “Low Levels of Respiratory Syncytial Virus Activity in Europe During the 2020/21 Season: What Can we Expect in the Coming Summer and Autumn/Winter?” Euro Surveillance 26 (2021): 2100639. 34296672 10.2807/1560-7917.ES.2021.26.29.2100639 PMC8299745

[32] E. K. Broberg , M. Waris , K. Johansen , P. Penttinen , and European Influenza Surveillance Network , “Seasonality and Geographical Spread of Respiratory Syncytial Virus Epidemics in 15 European Countries, 2010 to 2016,” Euro Surveillance 23 (2018): 17–284. 10.2807/1560-7917.ES.2018.23.5.17-00284 PMC5801642 29409569

[33] P. Obando‐Pacheco , A. J. Justicia‐Grande , I. Rivero‐Calle , et al., “Respiratory Syncytial Virus Seasonality: A Global Overview,” The Journal of Infectious Diseases 217 (2018): 1356–1364. 29390105 10.1093/infdis/jiy056

[34] S. Jiménez‐Jorge , C. Delgado‐Sanza , S. de Mateo , et al., “Monitoring Respiratory Syncytial Virus Through the Spanish Influenza Surveillance System, 2006–2014,” Enfermedades Infecciosas Y Microbiología Clínica 34 (2016): 117–120. 25703209 10.1016/j.eimc.2014.12.012

[35] T. Shi , A. Denouel , A. K. Tietjen , et al., “Global Disease Burden Estimates of Respiratory Syncytial Virus‐Associated Acute Respiratory Infection in Older Adults in 2015: A Systematic Review and Meta‐Analysis,” The Journal of Infectious Diseases 222 (2020): S577–S583. 30880339 10.1093/infdis/jiz059

[36] J. S. Nguyen‐Van‐Tam , M. O'Leary , E. T. Martin , et al., “Burden of Respiratory Syncytial Virus Infection in Older and High‐Risk Adults: A Systematic Review and Meta‐Analysis of the Evidence From Developed Countries,” European Respiratory Review 31 (2022): 220105. 36384703 10.1183/16000617.0105-2022 PMC9724807

[37] E. A. F. Simões , L. Bont , P. Manzoni , et al., “Past, Present and Future Approaches to the Prevention and Treatment of Respiratory Syncytial Virus Infection in Children,” Infectious Disease and Therapy 7 (2018): 87–120. 10.1007/s40121-018-0188-z PMC5840107 29470837

[38] AstraZeneca , “Beyfortus Approved in the EU for the Prevention of RSV Lower Respiratory Tract Disease in Infants 2022,” accessed January 19, 2023, https://www.astrazeneca.com/media‐centre/press‐releases/2022/beyfortus‐approved‐in‐the‐eu‐for‐the‐prevention‐of‐rsv‐lower‐respiratory‐tract‐disease‐in‐infants.html.

[39] A. G. Jansen , E. A. Sanders , A. W. Hoes , A. M. van Loon , and E. Hak , “Influenza‐ and Respiratory Syncytial Virus‐Associated Mortality and Hospitalisations,” The European Respiratory Journal 30 (2007): 1158–1166. 17715167 10.1183/09031936.00034407

[40] A. D. Colosia , J. Yang , E. Hillson , et al., “The Epidemiology of Medically Attended Respiratory Syncytial Virus in Older Adults in the United States: A Systematic Review,” PLoS ONE 12 (2017): e0182321. 28797053 10.1371/journal.pone.0182321 PMC5552193

[41] C. L. Hansen , S. S. Chaves , C. Demont , and C. Viboud , “Mortality Associated With Influenza and Respiratory Syncytial Virus in the US, 1999–2018,” JAMA Network Open 5 (2022): e220527. 35226079 10.1001/jamanetworkopen.2022.0527 PMC8886548

[42] M. Haeberer , R. Bruyndonckx , A. Polkowska‐Kramek , et al., “Estimated Respiratory Syncytial Virus‐Related Hospitalizations and Deaths Among Children and Adults in Spain, 2016–2019,” Infectious Disease and Therapy 13 (2024): 463–480. 10.1007/s40121-024-00920-7 PMC10965866 38319540

[43] N. Torner , E. Navas , N. Soldevila , et al., “Costs Associated With Influenza‐Related Hospitalization in the Elderly,” Human Vaccines & Immunotherapeutics 13 (2017): 412–416. 27925855 10.1080/21645515.2017.1264829 PMC5328227

[44] J. Díez‐Domingo , E. Redondo Margüello , R. Ortiz de Lejarazu Leonardo , et al., “A Tool for Early Estimation of Influenza Vaccination Coverage in Spanish General Population and Healthcare Workers in the 2018–19 Season: The Gripometro,” BMC Public Health 22 (2022): 825. 35468772 10.1186/s12889-022-13193-x PMC9036844

[45] P. Jorgensen , J. Mereckiene , S. Cotter , K. Johansen , S. Tsolova , and C. Brown , “How Close Are Countries of the WHO European Region to Achieving the Goal of Vaccinating 75% of Key Risk Groups Against Influenza? Results From National Surveys on Seasonal Influenza Vaccination Programmes, 2008/2009 to 2014/2015,” Vaccine 36 (2018): 442–452. 29287683 10.1016/j.vaccine.2017.12.019 PMC5777640

