# Supplementary material for: How Does the Burden of Respiratory Syncytial Virus Compare to Influenza in Spanish Adults?
Source: Influenza Other Respir Viruses. 2024 Jun 24;18(6):e13341. doi: 10.1111/irv.13341 (PMC11194680; doi:10.1111/irv.13341)
Supplement: Supplementary file 1 — FIGURE S1 Statistical projection method to assess the potential of hospitals to be excluded from this study. TABLE S1: Diagnostic codes used to identify RSV patients. TABLE S2: Diagnostic codes used to identify influenza patients. TABLE S3: Other procedure codes used to identify cases of interest. TABLE S4: Diagnostic codes used to identify predefined comorbidities. TABLE S5: Respiratory diagnoses (for analyses of respiratory readmission < 30 days after RSV/influenza A admission discharge). TABLE S6: Diagnostic codes used to identify complications. TABLE S7: Complications in patients infected with respiratory syncytial virus or influenza stratified by age group in Spain (2015, 2017, and 2018). [file IRV-18-e13341-s001.docx]

**How does the burden of respiratory syncytial virus compare to influenza in Spanish adults?**

**Short running title: RSV vs influenza burden of hospitalization – Spain**

Federico Martinón-Torres^1,2,3^, Clara Gutierrez^4^, Ana Cáceres^4^, Karin Weber^5^ and
Antoni Torres^3,6,7,8^

^1^Translational Pediatrics and Infectious Diseases, Hospital Clínico Universitario de Santiago de Compostela, Santiago de Compostela, Spain. ^2^Genetics, Vaccines and Pediatric Infectious Diseases Research Group, Instituto de Investigación Sanitaria de Santiago, Universidad de Santiago, Santiago de Compostela, Spain. ^3^Consorcio Centro de Investigación Biomédica en Red de Enfermedades Respiratorias (CIBERES), Instituto de Salud Carlos III, Madrid, Spain. ^4^Janssen-Cilag, Madrid, Spain. ^5^Global Medical Affairs IDV, Janssen-Cilag, Vienna, Austria. ^6^Dept of Pneumonology, Hospital Clinic of Barcelona, Barcelona, Spain. ^7^August Pi i Sunyer Biomedical Research Institute (IDIBAPS), Barcelona, Spain. ^8^ICREA Academia, Life and Medical Sciences, Universitat de Barcelona, Barcelona, Spain.

**Correspondence to:**

Federico Martinón-Torres

UCI Pediatria. Hospital Clínico Universitario de Santiago de Compostela.

A Choupana.s.n.

15071 Santiago de Compostela (Spain)

[Federico.martinon.torres@sergas.es](mailto:Federico.martinon.torres@sergas.es)

**SUPPLEMENTARY APPENDIX**

**FIGURE S1:** Statistical projection method to assess the potential of hospitals to be excluded from this study.

**
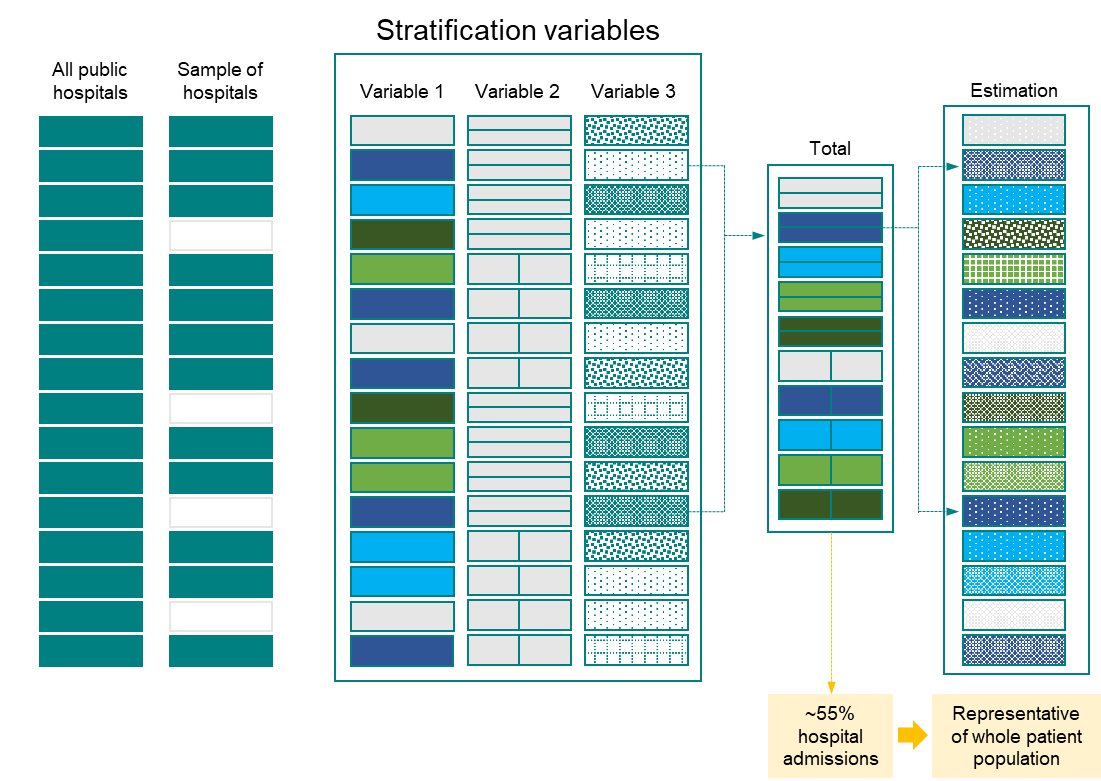
**

The MSU is the stratification unit with a minimum number of hospitals required for calculating the estimates. The number of hospitals that make up the MSU depends on the total number of centres across the country. For this study, it is assumed that working with an MSU consisting of 3 hospitals is feasible.

MSU, minimum stratification unit.

**TABLE S1:** Diagnostic codes used to identify RSV patients.

| **Description** | **ICD-9-CM** | **ICD-10-ES** |
| --- | --- | --- |
| **RSV** | 079.6 | NA |
| **Acute bronchiolitis due to RSV** | 466.11 | [J21.0](https://www.icd10data.com/ICD10CM/Codes/J00-J99/J20-J22/J21-/J21.0) |
| **Pneumonia due to RSV** | 480.1 | [J12.1](https://www.icd10data.com/ICD10CM/Codes/J00-J99/J09-J18/J12-/J12.1) |
| **RSV as the cause of diseases classified elsewhere** | NA | B97.4 |
| **Acute bronchitis due to RSV** | NA | J20.5 |

NA, not applicable; RSV, respiratory syncytial virus.

**TABLE S2:** Diagnostic codes used to identify influenza patients.

| Description | ICD-9-CM | ICD-10-ES |
| --- | --- | --- |
| Influenza with pneumonia | 487 | NA |
| Influenza with other respiratory manifestations (acute upper respiratory infections, laryngitis, pharyngitis, pleural effusion) | 487.1 | J10.1 |
| Influenza with other manifestations (gastrointestinal, acute myocarditis) | 487.8 | NA |
| Influenza due to identified avian influenza virus A/H5N1 with pneumonia | 488.01 | NA |
| Influenza due to identified avian influenza virus A/H5N1 with other respiratory manifestations (acute upper respiratory infections, laryngitis, pharyngitis, pleural effusion) | 488.02 | NA |
| Influenza due to identified avian influenza virus A/H5N1 with other manifestations (gastrointestinal, acute myocarditis, encephalopathy etc.) | 488.09 | NA |
| Influenza due to identified influenza A (H1N1) pdm09 with pneumonia | 488.11 | NA |
| Influenza due to identified influenza A (H1N1) pdm09 with other respiratory manifestations (acute upper respiratory infections, laryngitis, pharyngitis, pleural effusion) | 488.12 | NA |
| Influenza due to identified influenza A (H1N1) pdm09 with other manifestations (gastrointestinal, acute myocarditis, encephalopathy etc.) | 488.19 | NA |
| Influenza due to novel influenza A (excludes avian influenza, influenza A/H5N1, influenza A (H1N1) pdm09) | 488.8 | NA |
| Influenza due to certain identified influenza viruses |  | J09 |
| Influenza due to identified novel influenza A virus with pneumonia |  | J09.X1 |
| Influenza due to identified novel influenza A virus with other respiratory manifestations |  | J09.X2 |
| Influenza due to identified novel influenza A virus with gastrointestinal manifestations |  | J09.X3 |
| Influenza due to identified novel influenza A virus with other manifestations |  | J09.X9 |
| Influenza due to other identified influenza virus with unspecified type of pneumonia |  | J10.00 |
| Influenza due to other identified influenza virus with the same other identified influenza virus pneumonia |  | J10.01 |
| Influenza due to other identified influenza virus with other specified pneumonia |  | J10.08 |
| Influenza due to another identified influenza virus with gastrointestinal manifestations |  | J10.2 |
| Influenza due to other identified influenza virus with encephalopathy |  | J10.81 |
| Influenza due to other identified influenza virus with myocarditis |  | J10.82 |
| Influenza due to other identified influenza virus with otitis media |  | J10.83 |
| Influenza due to other identified influenza virus with other manifestations |  | J10.89 |

NA, not applicable.

**TABLE S3:** Other procedure codes used to identify cases of interest.

| **Definition** | **ICD-9-CM** | **ICD-10-ES** |
| --- | --- | --- |
| **Invasive mechanical ventilation** | 96.7x |  |

**TABLE S4:** Diagnostic codes used to identify predefined comorbidities.

| **Broader** | **Narrow** |  | **ICD-9-CM** | **ICD-10-ES** |  |
| --- | --- | --- | --- | --- | --- |
|  |  |  |  |  |  |
| **Respiratory/** | Bronchiectasis |  | 748.61, 494.0, 494.1, 011.5 | J47.0, J47.1, J47.9, Q33.4 |  |
| **lung** | Asthma |  | 493.xx | J45.20, J45.21, J45.22, J45.30, J45.31, J45.32, J45.40, J45.41, J45.42, J45.50, J45.51, J45.52, J45.901, J45.902, J45.909, J45.991, J45.998 |  |
|  | COPD |  | 490-492.x; 496 | J43.0, J43.1, J43.2, J43.8, J43.9, J44.0, J44.1, J44.9 |  |
|  | Post-inflammatory pulmonary fibrosis, chronic and other pulmonary manifestations due to radiation, chronic respiratory disease arising in the perinatal period, idiopathic fibrosing alveolitis, with pulmonary manifestations |  | 515, 508.1, 770.7, 516.3, 277.02 | J84.10, J70.1, P27, J84.112, E84.0 |  |
|  | Rheumatoid lung |  | 714.81 | M05.1 |  |
| **Cardiovascular** | Hypertensive disease |  | 401-40 | I11.0, I11.9, I16.0, I16.1, I16.9 |  |
|  |  |  |  |  |  |
|  | Other disease of pericardium |  | 423 | I30, I31, I32 |  |
|  | Other diseases of endocardium |  | 424 | I33 |  |
|  | Cardiomyopathy |  | 425 | I41, I42, I43 |  |
|  | Conduction disorders |  | 426 | I45, I46 |  |
|  | Cardiac dysrhythmias |  | 427 | I49 |  |
|  | Heart failure |  | 428 | I50.1, I50.8, I50.9 |  |
|  | Ill-defined descriptions and complications of heart disease |  | 429 | I51 |  |
|  | CVD |  | 430 - 438.xx | I60-I69; G45-G46; |  |
|  | Ischaemic heart disease |  | 410.xx - 414.xx | A18.84; I20; I21 (excl. I21.9); I22, I24-128, I34-I39; M32.11; M32.12; T80.0; T81.71; T81.72; T82.81 |  |
|  | Peripheral vascular disease |  | 443.9 | I73.9 |  |
|  | High risk congenital heart disease |  | 425.4; 428.0; 745.0 - 745.4; 745.6x - 745.8; 746.01 - 746.5; 746.7 - 746.85; 746.87; 747.1x; 747.21 -747.49 | I50.2; I50.3; I50.4; I50.9; Q20-Q26 |  |
|  | Low risk congenital heart disease |  | 745.5; 745.9; 746.00; 746.6; 746.86; 746.89; 746.9; 747.0; 747.20; 747.83 | P29.3; Q20.9; Q21.1; Q21.9; Q22.3; Q23.3; |  |
|  |  |  |  | Q23.8; Q23.9; Q24.6; Q24.8; Q24.9; Q25.0; Q25.4 |  |
| **Immuno-compromised** | HSCT |  | 996.88, 996.85, 41.04 - 41.09 | T86.5. |  |
|  | Lung transplant |  | 996.84; V42.6, 32.3x - 32.5x; 33.5x | Z94.2, T86.83 |  |
|  |  |  |  | T86.81, |  |
|  | HIV |  | 042.xx, | B20 |  |
|  | Hematologic malignancy (leukaemia, lymphoma, multiple myeloma) |  | 203.xx - 208.xx; 238.4; 238.72 - 238.76; 289.83 | C81-C96 |  |
|  |  |  |  |  |  |
|  | Non-lung solid organ transplant |  | 199.2; 996.52; 996.55; 996.80 - 996.83; 996.86 - 996.89; E878.0; V42.0 - V42.3; V42.7; V42.83; V42.84; V45.87; V58.44, 07.94; 37.51; 41.94; 46.97; 50.51; 50.59; 52.80; 52.82; 52.83; 55.53; 55.69 | Z94.0, Z94.1, Z94.4, Z94.5, Z94.7, T86.1, T86.2, T86.4, Z98.85, Z48.288, Z48.298, |  |
| **Chronic kidney disease** |  |  | 403.00, 403.1, 585.1, 585.2, 585.3, 585.4, 585.5, 585.6, 585.9 | I12.0, I12.9, N18.1, N18.2, N18.3, N18.4, N18.5, N18.6, N18. |  |

COPD, chronic obstructive pulmonary disease; HIV, human immunodeficiency virus; HSCT, hematopoietic stem cell transplantation.

**TABLE S5:** Respiratory diagnoses (for analyses of *respiratory* readmission <30 days after RSV/influenza A admission discharge).

| Definition | ICD-9-CM | ICD-10-ES |
| --- | --- | --- |
| BPD | 770.7x | P27.1 |
| Asthma | 493.x | J45.20, J45.21,J45.22, J45.30, J45.31, J45.32,J45.40, J45.41, J45.42, J45.50, J45.51, J45.52, J45.901, J45.902, J45.909, J45.991, J45.998 |
| Pneumonia (including pneumonia due to RSV and influenza) | 480.xx-487.xx, 488.01, 488.11, 488.81, 011.6x 516.3x, 517.1 | J12-18 |
| COPD | 490-492.x; 496 | J43.0, J43.1, J43.2, J43.8, J43.9, J44.0,  J44.1, J44.9 |
| Respiratory disease | 460-519.xx | J00-199 |
| Chronic pulmonary other metabolic and immunity disorders | 277.0x | E84 |
| COPD and allied conditions | 490-492.x; 496 | J43.0, J43.1, J43.2, J43.8, J43.9, J44.0, J44.1, J44.9 |
| Other diseases of respiratory system | 510.x, 513.x -517.x, 518.0 -518.3, 519.0x, 519.9 | J43, J84.84, J85, J91 |
| Tuberculosis | 011.xx, 012.xx | A15 |
| Diseases due to other mycobacteria | 031.0 | A31 |
| Sarcoidosis | 135 | D86.0, D86.2 |
| Ischemic heart disease (acute myocardial infarction, other acute and subacute forms of ischemic heart disease, old myocardial infarction angina pectoris) | 410-413 | I20-I24 |
| Other forms of heart disease (acute pericarditis acute myocarditis, acute/subacute endocarditis) | 420-424 | I30, I33, I38 - I40 |

BPD, bronchopulmonary dysplasia; COPD, chronic obstructive pulmonary disease; RSV, respiratory syncytial virus.

**TABLE S6:** Diagnostic codes used to identify complications.

| Definition | ICD-9-CM | ICD-10-ES |
| --- | --- | --- |
| Bacterial Pneumonia | 003.22, 041.3, 055.1, 073.0, 011.6X. 481, 482.0, 482.1, 482.2, 482.3X, 482.8X, 482.9, 483.XX, 483.4X, 484.XX, 495.7, 495.8, 495.9, 997.31 | A01.03, A02.22, J13, J14, J15, J16, J18 J95.851 |
| Acute kidney disease (acute glomerulonephritis, acute kidney failure, renal failure, unspecified) | 580, 584, 586 | N10, N17 |
| Exacerbation of chronic kidney disease | 403.00, 403.1, 585.1, 585.2, 585.3 585.4, 585.5, 585.6, 585.9 | I12.0, I12.9, N18.1, N18.2, N18.3, N18.4, N18.5, N18.6, N18 |
| Decompensation of a previously controlled diabetes | 249250 | E08, E10, E11, E13 |
| Myositis | 359.71, 376.12, 728.0, 728.11, 728.12, 728.81, 729.1 | M60.0, M60.1, M60.2, M60.8, M60.9, M61.0, M61.1, H05.12 |
| Pulmonary disease with exacerbation (obstructive chronic bronchitis with (acute) exacerbation, intrinsic/extrinsic asthma with (acute) exacerbation, asthma, unspecified type, with (acute) exacerbation, COPD with acute lower respiratory infection, COPD with (acute) exacerbation, unspecified asthma with (acute) exacerbation | 491.21, 493.02, 493.12, 493.22, 492.92, 494.1 | J44.0, J44.1, J45.901, J45.21, J45.31, J45.41, J45.51 |
| Respiratory failure | 581.81, 581.83, 518.84, 770.84 | J96.0, J96.1, J96.2, J96.9 |
| Rhabdomyolysis | 728.88 | M62.82 |
| Septic Shock | 785.52 | R65.21 |
| Severe dehydration | 276.51 | E86.0 |
| Ischemic heart disease (acute myocardial infarction, other acute and subacute forms of ischemic heart disease, old myocardial infarction, angina pectoris) | 410 - 413 | 120 - 124 |
| Other forms of heart disease (acute pericarditis, acute myocarditis, acute/subacute endocarditis), respiratory distress syndrome, other pulmonary insufficiency, not elsewhere classified | 420 – 423, 518.82 | I30, I33, I38 - I40, J80, P22.0 |

COPD, chronic obstructive pulmonary disease.

**TABLE S7:** Complications in patients infected with respiratory syncytial virus or influenza stratified by age group in Spain (2015, 2017 and 2018)

|  | Bacterial pneumonia,  n (%) | Acute kidney disease,  n (%) | Exacerbation of chronic kidney diseases,  n (%) | Decompen-sation of a previously-controlled diabetes,  n (%) | Myositis,  n (%) | Pulmonary disease with exacerbation, n (%) | Respiratory failure,  n (%) | Rhab- domyolysis, n (%) | Septic shock,  n (%) | Severe dehydration,  n (%) | Ischaemic heart disease,  n (%) | Other forms  of heart disease,  n (%) |
| --- | --- | --- | --- | --- | --- | --- | --- | --- | --- | --- | --- | --- |
| RSV |  |  |  |  |  |  |  |  |  |  |  |  |
| 18–50 years | 15  (9.6) | 19  (12.2) | 24  (15.4) | 2 (1.3) | 0 | 36  (23.1) | 49 (31.4) | 3 (1.9) | 2 (1.3) | 0 | 3 (1.9) | 3 (1.9) |
| 51–64 years | 25  (4.8) | 46  (8.8) | 69  (13.2) | 15 (2.9) | 0 | 111 (21.3) | 226 (43.3) | 1 (0.2) | 7 (1.3) | 1 (0.2) | 21 (4.0) | 0 |
| 65–74 years | 43  (5.2) | 83  (10.1) | 83 (10.1) | 33 (4.0) | 0 | 199 (24.3) | 326 (39.8) | 2 (0.2) | 11 (1.3) | 9 (1.1) | 25 (3.0) | 6 (0.7) |
| 75–84 years | 51  (3.4) | 162  (10.7) | 245 (16.2) | 68 (4.5) | 0 | 289 (19.2) | 541  (35.9) | 0 | 20 (1.3) | 9 (0.6) | 116 (7.7) | 7 (0.5) |
| ≥85 years | 47  (2.3) | 270  (13.5) | 420 (20.9) | 73 (3.6) | 0 | 212 (10.6) | 812 (40.5) | 2  (0.1) | 2 (0.1) | 19 (0.9) | 148 (7.4) | 1  (0.1) |
| Total | **181  (3.6)** | **580  (11.6)** | **841 (16.8)** | **191 (3.8)** | **0** | **847 (16.9)** | **1,954 (39.0)** | **8 (0.2)** | **42 (0.8)** | **38 (0.8)** | **313 (6.2)** | **17 (0.3)** |
| Influenza |  |  |  |  |  |  |  |  |  |  |  |  |
| 18–50 years | 56 (5.8) | 19 (2.0) | 106 (10.9) | 20 (2.1) | 4 (0.4) | 319 (32.8) | 324 (33.3) | 4 (0.4) | 32  (3.3) | 8  (0.8) | 54 (5.5) | 27 (2.8) |
| 51–64 years | 170 (6.6) | 46 (1.8) | 286 (11.1) | 95 (3.7) | 0 | 661 (25.7) | 1,079 (41.9) | 16 (0.6) | 75 (2.9) | 24 (0.9) | 95 (3.7) | 29 (1.1) |
| 65–74 years | 180 (4.4) | 83 (2.0) | 597 (14.6) | 118 (2.9) | 4 (0.1) | 955 (23.4) | 1,729 (42.3) | 25 (0.6) | 102 (2.5) | 24 (0.6) | 240 (5.9) | 28 (0.7) |
| 75–84 years | 268 (4.0) | 162 (2.4) | 1,378 (20.6) | 260 (3.9) | 5  (0.1) | 1,312 (19.6) | 2,548 (38.2) | 62 (0.9) | 89 (1.3) | 66 (1.0) | 503 (7.5) | 24 (0.4) |
| ≥85 years | 308 (3.7) | 270 (3.3) | 2,201 (26.8) | 258 (3.1) | 0 | 976 (11.9) | 3,334 (40.6) | 43 (0.5) | 51 (0.6) | 169 (2.1) | 599 (7.3) | 9 (0.1) |
| Total | **982 (4.4)** | **580 (2.6)** | **4,568 (20.3)** | **751 (3.3)** | **13 (0.1)** | **4,223 (18.7)** | **9,014 (40.0)** | **150 (0.7)** | **349 (1.5)** | **291 (1.3)** | **1,491 (6.6)** | **117 (0.5)** |

Data have been calculated as a percentage of the total number of reported complications in each age category.

RSV, respiratory syncytial virus.
